# Control of NK Cell Activation by Immune Checkpoint Molecules

**DOI:** 10.3390/ijms18102129

**Published:** 2017-10-12

**Authors:** Asma Beldi-Ferchiou, Sophie Caillat-Zucman

**Affiliations:** 1 Laboratoire d’Immunologie, Hôpital Henri Mondor, Assistance Publique–Hôpitaux de Paris, 94000 Créteil, France; asma.ferchiou@aphp.fr; 2 Centre de Recherches sur l’Inflammation, Institut National de Recherche Médicale (INSERM) UMR1149, Université Paris Diderot, 75013 Paris, France; 3 Laboratoire d’Immunologie, Hôpital Saint-Louis, Assistance Publique–Hôpitaux de Paris, 75010 Paris, France

**Keywords:** NK cells, immune checkpoint molecules, cancer immunotherapy

## Abstract

The development of cancer and chronic infections is facilitated by many subversion mechanisms, among which enhanced expression of immune checkpoints molecules, such as programmed death-1 (PD-1) and cytotoxic T lymphocyte-associated antigen 4 (CTLA-4), on exhausted T cells. Recently, immune checkpoint inhibitors have shown remarkable efficiency in the treatment of a number of cancers. However, expression of immune checkpoints on natural killer (NK) cells and its functional consequences on NK cell effector functions are much less explored. In this review, we focus on the current knowledge on expression of various immune checkpoints in NK cells, how it can alter NK cell-mediated cytotoxicity and cytokine production. Dissecting the role of these inhibitory mechanisms in NK cells is critical for the full understanding of the mode of action of immunotherapies using checkpoint inhibitors in the treatment of cancers and chronic infections.

## 1. Introduction

Natural killer (NK) cells are key players in the elimination of cells that have undergone infection, malignant transformation, or even physical or chemical damage [1,2,3,4,5]. In contrast to T or B lymphocytes, reactivity of NK cells toward their targets does not require prior sensitization and is not dependent on a single dominant receptor. Actually, NK cells are equipped with a large repertoire of germline-encoded activating and inhibitory receptors [1,4,6,7,8,9]. Integration of all signals transmitted by these receptors tightly regulates NK-cell behavior and ultimately determines the magnitude of NK-cell-mediated cytotoxicity and cytokine production [7,8,9].

Inhibitory receptors such as killer cell Ig-like receptors (KIRs) and natural killer cell receptor group 2 member A CD94/NKG2A heterodimer recognize major histocompatibility complex (MHC) class I molecules. Since these molecules are ubiquitously expressed on most healthy normal cells, their interaction with NK-cell inhibitory receptors ensures that NK cells are kept in calm in physiological condition. Consequently, cells with reduced MHC class I expression, a situation frequently observed during the course of tumors or viral infections, do not provide enough inhibitory signals and thus, become sensitive targets for NK-cell mediated killing [10,11,12]. To become fully competent, NK cells undergo an education process during their development to ensure that only those that successfully engage their inhibitory receptors with the cognate host’s MHC class I molecules become functionally mature. This kind of “central” tolerance mechanism sets the triggering threshold of individual NK cells in order to prevent reactivity against self [10,13,14].

Beside inhibitory receptors, NK cells express panoply of activating receptors that recognize a large spectrum of ligands usually absent from the surface of healthy cells, such as tumor/viral-derived proteins or stress-induced molecules. Upon engagement by their cognate ligands, NK cell activating receptors trigger target cell lysis and release of pro-inflammatory cytokines (IFN-γ, TNF-α) [4,6,15,16]. NK cells are also equipped with the CD16 molecule (FcγRIIIA), which allows Antibody-dependent cellular cytotoxicity (ADCC) upon recognition of IgG antibody-coated target cells. Depending on their relative surface expression of the CD56 and CD16 molecules, NK cells are distinguished into two major subsets, CD56^bright^ CD16^−^ cells (around 10% of peripheral blood NK cells) and the most mature CD56^dim^ CD16^+^ cell subset. These two subsets are associated with different expression of some receptors, in particular KIR and CD94/NKG2A, and distinct functional capabilities [17,18,19].

NK cells are not only “killer cells”, albeit they were originally discovered thanks to their ability to spontaneously kill tumor cells. Indeed, through their ability to produce various soluble factors, NK cells interact with other immune cells and help promoting the development of efficient adaptive immune responses [20,21,22].

Thanks to their intrinsic properties, NK cells have entailed growing interest as promising therapeutic strategies to enhance immune surveillance in patients with cancer and infectious diseases. As such, their usage is already effective in the field of hematopoietic malignancies [23,24,25]. Accumulating evidence show that defects in NK cell function or number are associated with an increased susceptibility to develop viral infections and cancer [26,27]. In some cancers, quantitative NK-cell deficiency correlates with poor clinical outcomes [28]. Moreover, the development of chronic infections and cancers is facilitated by various immune subversion mechanisms targeting NK cell effector functions, such as the production of regulatory cytokines or immunosuppressive factors, decreased expression of activating receptors or their ligands, and expression of immune checkpoint molecules [29,30,31,32,33,34].

Immune checkpoint molecules are proteins that help keep immune responses in check, and thus can prevent immune cells, in particular T cells, from killing cancer cells. When the immune checkpoints are blocked, the brakes on the immune response are released, and T cells become able to kill cancer cells. Recently, targeting immune checkpoints with specific inhibitor antibodies has revolutionized the treatment of many cancers [35,36,37]. The main goal of such therapeutic strategies is to reverse exhaustion of T cells and reinvigorate their functional capacities. While enhanced expression of immune checkpoints such as programmed death-1 (PD-1), cytotoxic T lymphocyte-associated antigen 4 (CTLA-4), T cell immunoglobulin- and mucin-domain 3 (TIM-3), and lymphocyte activation gene-3 (LAG-3) has been largely demonstrated to decrease T cell functions, their expression and functional consequences in NK cells are much less explored. In this review, we focus on the current state of the art on expression of immune checkpoint molecules on NK cells and how it can interfere with NK cell functions. We also discuss how KIRs and CD94/NKG2A inhibitory receptors, although not classically defined as immune checkpoint molecules, modulate the duration and magnitude of NK cell responses and thus, recently emerged as promising targets for the treatment of cancer. For purpose of clarity, a summary of the immune checkpoint molecules, their expression and cognate ligands, and their major impact on NK cell functions is given in Table 1.

## 2. Programmed Cell Death-1

PD-1 is an immune checkpoint inhibitory receptor expressed on activated T and B lymphocytes. PD-1 binds the PD-L1 and PD-L2 ligands, which are expressed on tumors, infected cells, and on antigen presenting cells present in inflammatory infiltrates [38,39,40,41]. PD-1 is involved in terminating immune responses of antigen-stimulated T and B cells, thus contributing to the normal autoregulatory machinery. PD-1 regulates immune tolerance, as indicated by the occurrence of autoimmune diseases in PD-1 deficient mice [42,43] and by the association of PD-1 polymorphisms with susceptibility to various human autoimmune diseases [44,45,46]. Moreover, autoimmune manifestations are frequently observed as a side effect of PD-1 blockade strategies in the clinic [47,48]. In antigen-stimulated T cells, binding of PD-1 to its ligands leads to abrogation of the T cell receptor (TCR) signaling and subsequent inhibition of T cell functions [49,50,51,52]. Thus, the PD-1 axis plays an important role in limiting antigen-specific responses. This protective mechanism is frequently exploited by tumors and viruses to escape immune response. After repeated TCR stimulation during chronic viral infection or in the cancer microenvironment, PD-1 is persistently upregulated on virus- or tumor-specific T cells. Consequently, PD-1 receptor/ligand interactions hamper T-cell activation and effector functions, and ultimately result in T lymphocyte exhaustion [53,54]. Importantly, blockade of the PD-1 pathway can rescue PD-1^+^ T cells from exhaustion and reinvigorate their effector capacities. Therapeutic strategies that target the PD-1/PD-L1 interactions have recently shown remarkable clinical responses in patients with various cancer types [36,55,56,57]. The efficiency of such therapies is considered to largely rely on the enhanced effector functions of tumor-specific T cells.

In addition to T and B lymphocytes, expression of PD-1 was reported in innate or innate-like immune cells, such as NKT [58] cells and group 2 innate lymphoid cells (ILC-2) [59]. In NK cells, expression of PD-1 was reported in various tumor (renal cell carcinoma, multiple myeloma, ovarian carcinoma, Kaposi sarcoma or EBV-associated post-transplant lymphoproliferative disorder) and chronic infection (*M. tuberculosis*, Human cytomegalovirus (HCMV), Human Immunodeficiency Virus (HIV), or hepatitis C virus (HCV) infection) settings [30,60,61,62,63,64,65,66]. However, until recently, the phenotypic and functional characteristics of PD-1^+^ NK cells were not evaluated in depth. Recent studies demonstrate that, like in T cells, PD-1 is a critical negative regulator of NK cells. PD-1^+^ NK cells most often belong to the mature CD56^dim^ CD16^+^ population, but exhibit variable modifications of their activating or inhibitory receptor repertoire depending on the clinical setting [30,61,66]. Thus, PD-1 expression is confined to a fully mature/terminally differentiated NKG2A^−^KIR^+^CD57^+^ subpopulation in CMV-seropositive healthy subjects [66], but not in patients with Kaposi sarcoma [30]. In cancer patients, PD-1^+^ NK cells express markers of activation and apoptosis sensitivity, suggesting that they have experienced in vivo immune stimulation [30,60]. Such hypothesis is supported by in vitro experiments showing that PD-1 is induced upon prolonged stimulation by MHC class I-deficient tumor cells or by agonists of NK cell activating receptors [30,67], two conditions mimicking the tumor microenvironment. Notably, previous data demonstrated that NK cells are anergic in MHC-deficient tumor-bearing mice, although expression of PD-1 was not reported [68]. Irrespective of their phenotype, PD-1^+^ NK cells are functionally exhausted, exhibiting reduced proliferative capability, poor cytolytic activity and impaired cytokine production as compared with the PD-1^−^ NK cell population [30,61,66,67]. Moreover, in vitro blockade of the PD-1 pathway reverts the NK cell functional defects induced by PD-1/PDL-1 interactions, which confirms that PD-1 directly participates to NK cell exhaustion and is not simply a marker of chronic activation [61,66].

Altogether, these findings indicate that, like in T cells, PD-1 represents a mediator of functional exhaustion in NK cells and may serve to blunt NK cell-mediated antitumor response. Therefore, releasing the PD-1 immune checkpoint in NK cells might help to circumvent tumor escape by enhancing NK-cell trafficking and effector functions against the tumor, as shown for CD8 T cells. In this context, it will be crucial to determine if NK cell functions are improved in cancer patients treated with inhibitors of the PD-1 pathway, as addressed in an ongoing clinical trial aiming to assess the effect of pembrozilumab on NK cell exhaustion (NCT03241927, Clinical trial.gov).

## 3. Cytotoxic T Lymphocyte-Associated Antigen 4 (CTLA-4)

Cytotoxic T lymphocyte-associated antigen 4 (CTLA-4) is another major immune checkpoint molecule induced on T cells upon activation, and negatively regulating their ongoing functions [69]. To exert its inhibitory effect, CTLA-4 competes with CD28 for the same ligands, B7-1 and B7-2, expressed by antigen-presenting cells [70]. Its crucial role in maintaining immune homeostasis is substantiated by the development of aggressive and fatal autoimmune diseases in animal models lacking CTLA-4 [71,72]. Numerous studies have reported an increased and persistent expression of CTLA-4 on antigen-specific T cells during chronic viral infections and tumors [73,74]. CTLA-4 expression clearly contributes to perpetuate tumor immune escape by continuously inhibiting effector functions of tumor-infiltrating lymphocytes. However, data on CTLA-4 expression on NK cells are scarce. CTLA-4 is induced on mouse IL-2-activated NK cells [75] and is expressed on splenic Kit^+^ and tumor-infiltrating NK cells in tumor-bearing mice [75,76]. CTLA-4 may be directly involved in inhibiting NK-cell IFNγ production induced by mature dendritic cells [75]. Expression of CTLA-4 on human NK cells remains elusive, and the effect on NK cells of drugs targeting the CTLA-4 pathway in cancer patients has not been studied. It cannot be excluded that such therapeutic approaches could enhance NK cell effector function by an abscopal (out-of-target) effect. Indeed, anti-CTLA-4 therapy may target regulatory T (Treg) cells in vivo, thus unleashing NK cells from their suppressive effects [77,78]. Moreover, CTLA-4 blockade revivifies T cells and improves their IL-2 production [79]. In turn, IL-2 could ultimately reinvigorate NK-cell functions, especially when IL-2 becomes more available in the absence of Tregs [80]. Thus, one can speculate that anti-CTLA-4 therapy may also, at least indirectly, improve NK cell contribution to anti-tumor immune response.

## 4. T-Cell Immunoglobulin and Mucin Domain 3 (Tim-3)

T-cell immunoglobulin and mucin domain 3 (Tim-3) is a co-inhibitory receptor that was initially described on activated T cells [81,82]. Tim-3 is also expressed in other immune populations such as NK, NKT and myeloid cells. In addition to galectin-9 [81], Tim-3 has been reported to bind other ligands such as phosphatidylserine exposed by apoptotic cells [83], the alarmin HMGB1 [84], and the carcinoembryonic antigen cell adhesion molecule 1 (CEACAM1) [85]. Tim-3 is considered to play an important role in immune tolerance as a negative regulator of proinflammatory responses to avoid excessive host damage [86]. Of note, Tim-3^+^ Foxp3^+^ Tregs display a higher suppressive function than Tim3^−^ Foxp3^+^ Tregs [87]. Tim-3 is also involved in mediating T-cell exhaustion during cancer and chronic viral infections [88,89]. Sustained expression of both PD-1 and Tim-3 defines highly exhausted CD8 T cells, suggesting cooperation between these two receptors to continuously inhibit T cell functions [89]. In numerous tumor models, blocking Tim-3 pathway rescues T cells from exhaustion, restores their functions, and leads to a better control of tumor growth [90,91,92].

Expression of Tim-3 on NK cells is well established, but its consequences are controversial. NK cells from healthy donors express Tim-3 at steady state, mostly on the mature CD56^dim^ CD16^+^ NK cells subset [93,94]. This expression is strongly upregulated upon cytokine activation, especially after stimulation with IL-12 + IL-18 or IL-15 + IL-12 [93,94]. Thus, Tim-3 expression on NK cells appears as a marker of maturation and/or activation of NK cells [93,94,95]. However, the role of the Tim-3 pathway in NK cell functions is confusing, as it was assigned both activating and inhibitory effects. While stimulation with anti-Tim-3 agonist antibodies curtails NK cell cytotoxic potential [93], engagement of Tim-3 by its cognate ligand Gal-9 enhances IFNγ production but has no effect on NK cell cytotoxic ability [94]. Tim-3 blockade reduces NK cell-mediated killing of pancreatic cancer cell (PCC) lines [96]. However, in a context of chronic stimulation such as lung adenocarcinoma, advanced melanoma, or chronic hepatitis B, sustained Tim-3 expression defines a subset of functionally defective/exhausted NK cells, and Tim-3 blockade rescues NK cell functions [97,98,99]. Both Tim-3 and PD-1 are induced on mouse NK cells upon prolonged stimulation by MHC class I-deficient tumor cells. Tim-3^+^ PD-1^+^ NK cells are functionally exhausted, a defect reversible upon IL-21 stimulation [67].

Taken together, these data suggest that the role of Tim-3 in NK cells may vary according to the experimental or clinical setting. This is reminiscent of the dual role of another receptor, 2B4 (CD244), on NK cell activation. It is not excluded that in vivo, Tim-3 effect on NK cells depends on a functional threshold that fine-tunes the activity of Tim-3 signaling pathway. The promiscuous ligand specificity of Tim-3 may account for its controversial effect on NK cell function depending on the target cells considered, and/or the intensity and duration of Tim-3 receptor/ligand expression. The final effect may also depend on other factors including cytokine microenvironment. Thus, although manipulating the Tim-3 immune checkpoint on NK cells may appear promising to restore NK-cell mediated immune surveillance in cancer, further studies are warranted to better decipher Tim-3 effects in human NK cells.

## 5. T Cell Immunoglobulin and ITIM Domain (TIGIT)

TIGIT is an inhibitory receptor initially described on T and NK cells [100]. In NK cells, TIGIT competes with the DNAM-1 (CD226) activating receptor for their common ligands CD112 (PVRL2) and CD155 (PVR) expressed on many cancer cells [100,101,102,103,104,105]. TIGIT was shown to bind to its ligands with higher affinity than DNAM-1 [100,101,102]. TIGIT can directly dampen NK cell cytotoxicity and thus counterbalances the DNAM-1-mediated NK cell activation [101,102]. Recent data demonstrate that TIGIT sensitizes NK cells to immune suppression mediated by myeloid-derived suppressor cells (MDSC), an effect abrogated by TIGIT blockade [106]. Moreover, in vitro TIGIT blockade improves the anti-tumor effect of Trastuzumab (a recombinant humanized anti-HER2 monoclonal antibody used in HER2+ breast cancers), which partially relies on NK cell-mediated ADCC [105]. Thus, targeting the TIGIT axis may represent a promising additional tool to revivify NK cell functions in cancer patients.

## 6. Lymphocyte Activation Gene-3 (LAG-3)

LAG-3 is an inhibitory receptor presenting homologies with CD4 and can also bind to MHC class II molecules with higher affinity than CD4 [107,108,109]. LAG-3 expression was initially described on activated T and NK cells [109]. Further studies reported its expression in a wide range of immune cells including B cells, plasmacytoid dendritic cells, iNKT and Treg cells [110,111,112,113,114]. LAG-3 is able to directly inhibit T cell effector function [9,16,17,18] while it promotes Treg suppressive functions [110,112,115,116,117,118]. Accumulating data suggest that LAG-3 is involved in T cell exhaustion in various cancer and chronic infection settings [119,120]. In vitro LAG-3 blockade improves T cell functions [121], and the combined blockade of PD-1 and LAG-3 synergistically rescues T cells from exhaustion in many cancers and chronic infection models [122,123,124]. Importantly, several ongoing clinical trials involve the use of anti-LAG-3 antibodies either alone or in combination with PD-1 blockade in various cancers (ClinicalTrials.gov). The direct impact of LAG-3 on NK cells functions was rarely investigated. NK cells from LAG-3 deficient mice exhibit a decreased capacity to eliminate certain tumor cell lines [125]. However, data in human NK cells are controversial. Blocking the LAG-3 pathway by anti-LAG-3 antibodies or soluble LAG-3 (able to bind MHC class II) had no effect on NK cell cytotoxic capability, although cytokine production was not investigated in this study [126]. More recently, low NK cell expression of inhibitory molecules, including LAG-3, was found to be associated with viral control in HIV infected patients [127]. However, the direct impact of LAG-3 expression on NK cells functions was not investigated. So far, the contribution of LAG-3 in NK cell functions remains elusive.

## 7. Killer Cell Immunoglobulin-Like Receptors (KIRs)

NK cells constitutively express inhibitory KIRs that recognize determinants shared by groups of MHC class I alleles. During NK cell development, the expression KIRs is variegated, and diverse NK cell subsets displaying various combinations of KIRs emerge in a given individual. Upon engagement with their cognate MHC-I ligands, KIRs provide an inhibitory signal that prevents NK cell activation towards healthy autologous cells, which constitutively express MHC class I molecules. Diminished expression of MHC class I (upon tumor transformation or viral infection) renders cells highly sensitive to NK-cell mediated killing [4,128,129,130]. In other words, a compromised interaction between KIRs and the corresponding MHC class I molecules is unable to deliver sufficient inhibitory signals to NK cells, thus allowing their activation. Notably, interactions between KIRs and their cognate MHC class I ligands during NK development are critical for the education or licensing process of NK cells, by which NK cells adapt their responsiveness and achieve self-tolerance [13].

In the field of hematologic malignancies, haplo-identical stem cell transplantation from a KIR ligand-mismatched donor favors NK cell alloreactivity thanks to the lack of relevant KIR-HLA class I interaction [24,131]. Infusions of KIR ligand-mismatched allogeneic NK cells prior to autologous stem cell transplantation for relapsed multiple myeloma showed encouraging results [132]. These data suggest that disinhibited NK cells can exert their anti-tumor functions with superior efficacy, which is associated with increased patients’ survival.

Pharmacologic exploitation of inhibitory KIRs is possible and seems to be an attractive strategy to reinforce NK cell reactivity against tumor cells in order to achieve durable remission. Accumulating evidence point out the benefit of combining KIR blockade therapy to another immunotherapy relying on NK cell-mediated ADCC. For instance, in B cell lymphoma treatment, the efficacy of rituximab, an IgG1 anti-CD20 mAb, largely relies on NK cell-mediated ADCC [23,25,133]. However, FcγRIIIA polymorphism clearly influences rituximab-dependent NK cell cytotoxicity, and patients with FcγRIIIA polymorphism conferring a higher affinity for IgG1 have a better response rate [133,134]. A strategy to further improve rituximab responses is the use of KIR blockade therapy, which releases NK cells from the inhibition exerted by the high level of MHC-I expression on tumor cells. Ultimately, this strategy enhances rituximab-dependent cytotoxicity by NK cells [135]. Other mAbs therapies relying on NK cell-mediated ADCC could also benefit from such a combinatory strategy. Alternatively, combining KIR blockade with an immunomodulatory drug, such as lenalidomide known to enhance NK cell functions, could be of interest in the treatment of some hematologic malignancies such as multiple myeloma [136].

Anti-KIR therapy is already used (in combination with other agents) in several ongoing cancer clinical trials (Clinical trial.gov). Importantly, anti-KIR antibody administration seems to be well tolerated, with limited adverse effects [136,137]. Using pan-KIR blockade as a single anti-cancer therapy would probably not induce a sufficient clinical response, as shown in multiple myeloma [138]. Combinatory therapy seems to be a more promising choice.

## 8. Natural Killer Cell Receptor Group 2 Member A (NKG2A)

NKG2 lectin-like receptors are expressed as a heterodimer with C94 on NK cells and specifically recognize the non-classical MHC class I molecule, HLA-E. Upon interaction with HLA-E, CD94/NKG2A acts as an inhibitory receptor while its counter-receptor CD94/NKG2C delivers activating signals [139,140,141]. At contrast with classical MHC class I molecules, HLA-E binds a restricted subset of peptides derived from signal peptides of other MHC class I molecules including classical MHC class I and HLA-G [142]. Surface expression of HLA-E requires the presence of these peptides [143]. Therefore, expression level of HLA-E also mirrors the expression level of classical MHC class I molecules and HLA-G as well. Using CD94/NKG2A, NK cells indirectly monitor cell surface expression of both classical MHC class I molecules and HLA-G. Lack of HLA-E/NKG2A interaction triggers NK cell “missing-self” response. HLA-E is frequently upregulated on cells from many hematological malignancies or solid cancers [144,145,146]. Similar to KIR blockade, anti-NKG2A antibody can remove HLA-E-mediated NK cell inhibition, tip the balance to favor stimulation through NK cell activating receptors, and augment HLA-E accessibility for CD94/NKG2C. Monalizumab (IPH2201) is a therapeutic humanized IgG4 mAb that abrogates NKG2A receptor function. Monalizumab has already shown efficacy to strengthen NK cell responses against HLA-E overexpressing tumor cells in preclinical models [146], prompting its usage in various ongoing clinical trials including solid cancers and hematological malignancies (Clinical trial.gov).

## 9. Concluding Remarks

Dissecting the role of immune checkpoints in NK cells is crucial for the full understanding of the mode of action of therapies using checkpoint inhibitors. While several line of evidence indicates that PD-1 is a mediator of functional exhaustion in NK cells, the full understanding of the contribution of other immune checkpoints in the regulation of NK cell functions requires further investigation. It will be crucial to determine if unleashed NK cells help to improve anti-tumor response upon treatments with checkpoint inhibitors, but also if they may play a role in the inflammatory/autoimmune disorders that are associated with such treatments.

## Figures and Tables

**Table 1:** Overview of immune checkpoint molecules expressed on natural killer (NK) cells: pattern of expression, ligands, and impact on NK cell functions.

Inhibitory Immune Checkpoint	Expression	Ligands	Direct Impact on NK Cell Functions
PD-1	Activated T and B cells, NK cells, natural killer T (NKT) cells, ILC-2 cells and myeloid cells.	PD-L1 (B7-H1) and PD-L2 (B7-DC)	Inhibition of NK cell cytolytic activity and cytokine production.
CTLA-4	Treg cells, Activated T and B cells, Activated mouse NK cells, Human NK cells?	B7-1 and B7-2	Inhibition of mouse NK-cell IFN-γ production. Direct effect on human NK cells not documented.
TIM-3	NK, T, NKT and myeloid cells.	Galectin-9 HMGB1 CEACAM1	Dual role (inhibition/activation of NK cell functions) depending on the experimental or clinical setting.
T Cell Immunoglobulin and ITIM Domain (**TIGIT**)	NK cells, T cells.	CD112 (PVRL2) CD155 (PVR)	Inhibition of NK cell functions.
LAG-3	Activated T and NK cells, Treg, B cells, plasmacytoid dendritic cell (DC), NKT.	MHC class II molecules	The impact of LAG-3 on NK cell functions is controversial and not well documented.
Inhibitory KIRs	NK cells, CD8 T cells.	MHC class I molecules	Inhibition of NK cell functions.
NKG2A	NK cells, CD8 T cells.	HLA-E (non-classical MHC class I molecule)	Inhibition of NK cell functions.
